# Maze runners: monkeys show restricted Arabic numeral summation during computerized two-arm maze performance

**DOI:** 10.1007/s10071-024-01853-x

**Published:** 2024-03-06

**Authors:** Elizabeth L. Haseltine, Michael J. Beran

**Affiliations:** Psychology Department, Georgia State University, Atlanta, GA USA

**Keywords:** Mazes, Monkeys, Numerals, Summation, Inhibition, Individual differences

## Abstract

Mazes have been used in many forms to provide compelling results showcasing nonhuman animals’ capacities for spatial navigation, planning, and numerical competence. The current study presented computerized two-arm mazes to four rhesus macaques. Using these mazes, we assessed whether the monkeys could maximize rewards by overcoming mild delays in gratification and sum the values of Arabic numerals. Across four test phases, monkeys used a joystick controller to choose one of two maze arms on the screen. Each maze arm contained zero, one or two Arabic numerals, and any numerals in the chosen maze arm provided the monkeys with rewards equivalent to the value of those numerals. When deciding which arm to enter, monkeys had to consider distance to numerals and numeral value. In some tests, gaining the maximum reward required summing the value of two numerals within a given arm. All four monkeys successfully maximized reward when comparing single numerals and when comparing arms that each contained two numerals. However, some biases occurred that were suboptimal: the largest single numeral and the delay of reward (by placing numerals farther into an arm from the start location) sometimes interfered with the monkeys’ abilities to optimize. These results indicate that monkeys experience difficulties with inhibition toward single, high valence stimuli in tasks where those stimuli must be considered in relation to overall value when represented by symbolic stimuli such as numerals.

Symbolic stimuli are useful in presenting choice scenarios to humans in ways that let researchers observe what those participants emphasize as most relevant to their choices. Such stimuli can be combined with intertemporal choices that are presented as verbal problems (e.g., choosing between $20 after a time delay of 10 min or $50 after a time delay of 20 min). For humans, awareness of the symbolic meaning (e.g., $20 or $50) and of the time needed to get those rewards (e.g., 10 min or 20 min) allows an experimenter to present an almost limitless number of choice pairings. From these pairings, the relative importance of the delay to reward or the overall reward value (which could be presented as one lump sum or even a split payout over intervals) can be discovered through the choices that are made. Animals can be given similar choices, but typically this is through extensive experience of what a standard delay (or standard reward amount) is compared to an adjusting reward (or delay). These intertemporal choice tests are rarely symbolic for animals (see Logue 1988), although in some cases reward magnitude or delay length can be presented in analog form (e.g., Blanchard et al. 2013, 2015; Heilbronner and Hayden 2016). Unique trial combinations of reward and delay to reward are, therefore, hard to present in this context. In addition, such learning requirements often preclude asking whether two or more reward amounts can be combined in a way that accurately reflects their total value. Presenting animals with reward values that are symbolic (e.g., Arabic numerals), reinforced through intervals or as a lump sum, and with varying degrees of delay, will contribute to the understanding of what factors are most relevant to macaque decision making.

We know that some stimuli can come to have specific meanings for animals that approximate the representational value of those same stimuli for humans, such as Arabic numerals representing specific numbers of things. For example, we know that monkeys can choose single stimuli from pairs and even larger sets of numerals or other stimuli based on which individual stimulus denotes the largest reward values (e.g., Beran et al. 2008; Diester and Nieder 2010; Harris et al. 2007a, 2010; Livingstone et al. 2010; Washburn and Rumbaugh 1991). Chimpanzees can use Arabic numeral labels to indicate the number of items in arrays (Biro and Matsuzawa 2001; Boysen and Berntson 1989; Matsuzawa 1985), and grey parrots also can learn verbal symbols for numerosity (e.g., Pepperberg 2012). In all of these cases, the subjects seemingly treat those stimuli as if they represent specific numbers of items, although those representations appear to be somewhat “fuzzy” rather than exact cardinal values (e.g., Beran and Rumbaugh 2001).

In some cases, there is evidence that animals can engage in the summation of stimuli in spatially separable sets so that comparison among sets of things can occur (see Davis and Pérusse 1988). This is also true for analog stimuli such as when monkeys must sum the number of shapes across two pairs of choice stimuli or even sum the number of sides of two shapes in each pair of choice stimuli (e.g., Terrell and Thomas 1990). Other tasks involve animals summing the total amount of food found across two locations within choice options (e.g., Anderson et al. 2005; Beran 2001; Rugani et al. 2011; Rumbaugh et al. 1987). And, summation occurs in some species even for symbolic stimuli. For example, a chimpanzee showed that she could move through space to view two numerals, and then return to a start location and choose the numeral corresponding to the summed total (Boysen and Berntson 1989). Pepperberg (2012) also reported that a grey parrot could sum two visually presented Arabic numerals with the correct verbal label. These data again indicate the capacity for flexible responding to stimuli beyond just learning which stimulus within pairs is rewarded or rewarded more extensively. Although this capacity is not the same as performing formal arithmetic, it is likely related to that ability. Additional evidence of how flexibly such summation abilities can be integrated into other decision processes will inform us about the relative ease with which such summation occurs. If such summation requirements are presented in a context that also includes spatial information that acts as a proxy for delay to reward, it may become easier to ask animals whether they can sum symbolic stimuli and use the resulting summed values while considering other information (such as delay-to-reward).

Mazes offer the opportunity to assess various cognitive and behavioral capacities of species due to the time and distance manipulations that may affect choice when traversing a maze. Rather than being required to move their entire bodies through three dimensional mazes, computerized mazes have allowed animals to make pecks, finger movements, move joysticks, or engage in computerized tasks that mimic movement through a physical maze (e.g., Beran et al. 2015; Fragaszy et al. 2003, 2009; Iversen and Matsuzawa 2001; Menzel and Menzel 2007; Mushiake et al. 2001; Pan et al. 2011). These maze tasks have been highly influential in studying the spatial navigation and planning abilities of different species, mostly nonhuman primates, but some of which were non-primates (e.g., Miyata and Fujita 2008).

Manipulations of the maze allow the experimenter to control the number of choice options, the various goals to be obtained in the maze, and the complexity of movement in the maze. For example, to optimize reward within a maze requires understanding how each route may relate to reward type and amount, as a function of time or effort to complete the maze. Further, subjects must be capable of at least anticipating what might be found at the end of a row or arm based on past learning, memory, or even an arbitrary cue given to indicate the stimulus found at the end of a maze.

Research has shown that primates can maximize reward in mazes by making responses at choice points that sometimes involve moving away from goals (Beran et al. 2015; Harris et al. 2007a, 2010; Fragaszy et al. 2003, 2009; Mushiake et al. 2001; Pan et al. 2011). In some cases, they even can respond while using symbolic stimuli that represent how much reward is available at a specific spatial location within the maze. For example, Harris and Washburn (2005) reported that rhesus macaques could use the value of a numeral placed within a computerized maze to indicate how many times traveling to that numeral would be reinforced. This was evidenced by slower responding to those numerals when monkeys anticipated that the represented number of payoffs had already been given for that numeral. Harris et al. (2007b) extended this result by training rhesus monkeys to complete some number of runs through a computerized maze and then choose either an Arabic numeral that matched that number of runs or the letter D to indicate the runs were different from the numeral. Although not all monkeys could do this, one animal performed above chance levels.

In our present task, monkeys chose one of two maze arms containing Arabic numerals. Contact with any numeral led to that number of food rewards being immediately dispensed. Once a monkey committed to one arm, it could not go backwards in the maze. Thus, maximizing reward sometimes required looking at two or more numerals rather than only one (or only the first one). In addition, sometimes the greater total reward required choosing an arm that took longer to traverse before a numeral would be encountered, resulting in a mild delay of gratification. Again, monkeys have shown such delay tolerance in other computerized tasks (e.g., Evans and Beran 2014; Evans et al. 2014). This suggests monkeys might be capable of integrating such tolerance into a task where they may also need to sum the value of symbolic stimuli to maximize their total reward.

We first trained monkeys that Arabic numerals each led to a specific, different number of food rewards when contacted. The monkeys were moved to the first of four test conditions once they became proficient at choosing the large-valued numeral during times when only two options were presented, each placed within separate arms and at the same distance from the starting point.

To examine whether the monkeys could overcome mild delays in gratification while still maximizing reward represented by numerals, we used the length of our maze arms to produce several positions the stimuli could be contacted in, which resulted in varying lengths of time to reward. Due to previous research demonstrating monkeys’ abilities to understand symbolic ordinality (e.g., Beran et al. 2008) and overcome immediate gratification to receive larger rewards (e.g., Evans 2007), our first prediction was that the monkeys would travel farther distances in the maze to contact numerals that maximized their pellet reward.

In subsequent test conditions, some arms contained pairs of numerals. Here, we assessed the monkeys’ ability to compare the overall value of each arm through an approximate summation of stimuli (from two numerals being present in that arm) to maximize reward. Our second prediction was that the monkeys would still maximize reward but that there would be individual differences as seen in previous tasks involving summation in nonhuman primates (e.g., Evans et al. 2010). We also expected that certain features of trials might lead to more suboptimal responding from the perspective of maximizing reward. This includes instances where numerals presented closer in distance to the starting point might be disproportionately chosen (over numerals presented farther away), as might numerals representing larger individual food values (even when those numerals might be paired with small numbers that, when summed, did not lead to the greatest overall amount). These biases, although suboptimal, might indicate limitations in how monkeys can evaluate multiple pieces of information such as each value unit or the distance to contacting that unit.

## Methods

### Subjects

Four adult male rhesus macaques between the ages of 16 and 36 years were tested. All monkeys had many years of experience interacting with computerized testing systems and joystick controllers (see Richardson et al. 1990). Two monkeys, Lou and Murph, had previous experience in computerized tasks using Arabic numerals (Harris et al. 2007a, b, 2010), but the other two monkeys, Obi and Chewie, had no prior experience. The monkeys were singly housed, but some individuals had compatible social partners they were able to interact with daily in enclosures with indoor and outdoor access. Monkeys worked in their main indoor housing location where they were separated during testing but still had auditory and visual access to other monkeys at all times. Monkeys also had daily rest periods during which they could move to outdoor enclosures with climbing structures, natural substrate to walk on, and other forms of environmental enrichment. The monkeys were never food or water deprived, and participation in this task was voluntary. The current study was approved by Georgia State University’s Institutional Animal Care and Use Committee (IACUC), and the animal research program at Georgia State is accredited by the Association for Assessment and Accreditation of Laboratory Animal Care (AAALAC). All monkeys completed all phases except for the final phase (Test Phase 4) during which one monkey, Lou, was not available for testing due to circumstances unrelated to this project.

### Apparatus

All subjects were tested on individual computer systems which included 17-inch monitors with color display. Connected to each computer was a pellet dispenser that provided 45-mg banana-flavored pellets as food rewards. To interact with the program, monkeys were given joystick controllers that allowed them to navigate the maze and contact desired stimuli by hand manipulation of the joystick. The program software was written using Visual Basic 6.0.

### Design and procedure

*The computerized maze task*. In all phases of this experiment, a white screen was presented with a black two-arm maze onscreen. To navigate the maze and collect food rewards, monkeys had to use the joystick controller to move a red cursor onscreen from left to right through the selected arm. Within an arm, monkeys made contact with green squares containing an Arabic numeral and a solid-green square indicating the end of the maze. Numerals had values between 0 and 5 across all phases of the experiment. During all phases, once the monkeys made an arm choice, they were unable to go backwards in the maze to return to the unchosen arm. Upon reaching and contacting any Arabic numeral, the monkeys were immediately rewarded with the number of pellets equivalent to the value of the numeral. They could then continue farther through the arm, contacting any other numerals, if there were any, before coming to the green end point of the maze on the right side of the screen. After reaching the end point, they sometimes received an additional reward, depending on the phase of the experiment. If the maze was navigated perfectly, it could be completed in approximately 15–20 s across all testing phases. However, expected behaviors such as running into walls, not continuously moving, or taking time to consume rewards would all increase this duration. After a 2-s intertrial interval ended, another maze was presented. Monkeys worked on the task at their own pace, completing as many trials as they chose to complete during test sessions that ranged from 4 to 12 h when the computer program was available to the monkeys in their indoor enclosure. Water was always available during testing.

**Fig. 1:**
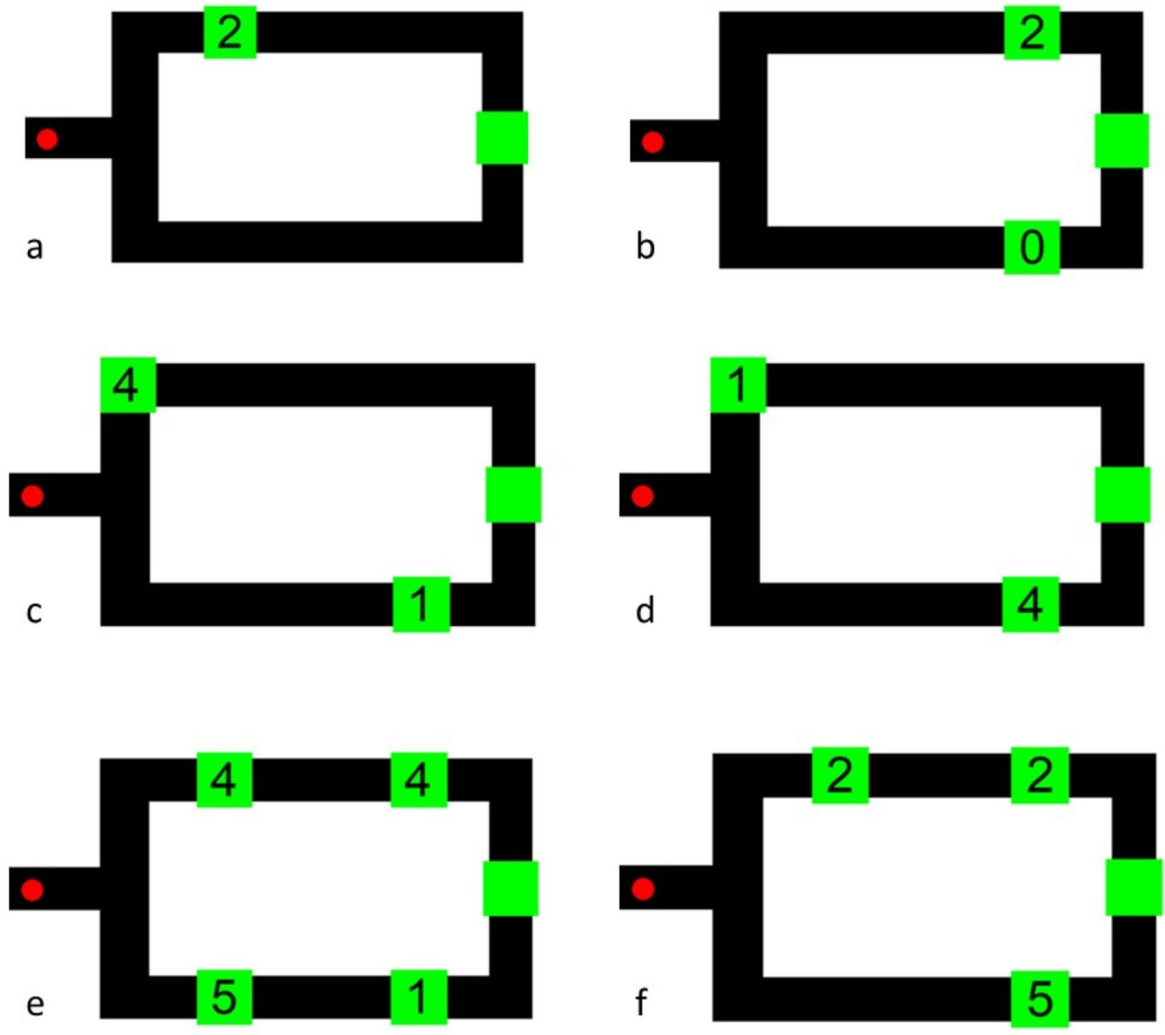
Example trial types from all phases. **a**. Training Phase 1; **b**. Training Phase 2 (also Baseline in Test Phase 1); **c**. Test Phase 1, Larger Numeral Closer; **d**. Test Phase 1, Larger Numeral Farther; **e**. Test Phase 2, First Numeral Smaller [Second Larger] for Arm with Larger Total; **f**. Test Phase 3 and 4, Lone Numeral Larger

*Training Phase 1 — Learning the value of numerals 1 through 5*. During the first training phase, one randomly selected Arabic numeral between 1 and 5 was placed on a surrounding green square and was located in one of the two maze arms at one of two distances from the starting point of the cursor (Fig. 1a). In this phase, if the monkeys selected the arm with a target numeral, they were rewarded with the number of pellets equivalent to the face value of the numeral, and they were given an additional pellet reward upon reaching the end point of the maze and contacting the green square located there. However, if they made an error by choosing the arm without a numeral in it, that would lead to no food rewards during or at the completion of the maze. Subjects had to choose the correct arm on 30 trials in this phase. These did not have to be consecutive trials.

*Training Phase 2 — Learning to maximize reward by choosing the larger single numeral*. During the second training phase, monkeys completed trials with the same two-arm maze but now there was an Arabic numeral in each arm (Fig. 1b). Both numbers were located the same distance from the starting point but in separate arms so that distance to a target numeral could not affect choice behavior. The numerals shown on a trial were never equal so that there was always a best choice in terms of maximizing food reward. After trials in which a monkey chose the smaller numeral, the next trial was a correction trial in which a zero was placed in the same arm that the monkey incorrectly chose on the previous trial, to aid in learning the numeral values and prevent side biases in responding. To successfully pass this training phase, individuals needed to be correct in choosing the larger numeral on 41 of the most recent 50 trials, not counting those correction trials. To start the testing phases, both training phases were required to be completed within a single session. If these prerequisites were not met in one session, a subject started with the first training phase on the next test session.

### Test phases

*Test Phase 1 — Comparing single numerals that vary in reward amount and distance*. Once training criteria were met, the monkeys were moved to the first test phase, which consisted of three trial types: Baseline, Larger Numeral Closer, and Larger Numeral Farther. Baseline trials were the same as the second training phase where two different numerals were presented the same distance away from the starting point (Fig. 1b). Larger Numeral Closer trials were those in which the larger numeral was placed closer to the start point than the smaller numeral, allowing the monkey to obtain the larger reward more quickly because it was closer (Fig. 1c). Larger Numeral Farther trials reversed these locations, with the larger numeral being farther down its respective arm from the start location than the smaller numeral (Fig. 1d). In this condition, it took longer to maximize reward and created a longer delay to receiving food. Duration of the delay was dependent on what position the numeral was located in the maze (positions 1 to 4). Between all positions, the approximate difference in reward retrieval time was multiple seconds (1-position difference [e.g., positions 1 and 2]: ~ 3 s; 2-position difference [e.g., positions 1 and 3]: ~ 7 s; 3-position difference [i.e., positions 1 and 4]: ~ 10 s. On trials where the two numerals were at different distances, the closer numeral was always in the top arm and the farther numeral in the bottom arm of the maze. Unlike the training phases, correct arm choices were not rewarded with an additional food reward at the end point of the maze. Contacting the green square at the end of the maze, which did not contain a numeral, simply served to end the trial and begin the 2 s intertrial interval. Each monkey completed 1,200 trials of this phase.

*Test Phase 2 — Maximizing reward by comparing the sum of numerals*. The purpose of Test Phase 2 was to assess how the monkeys responded when multiple numerals would be contacted in an arm, and particularly to see whether the monkeys could estimate which arm had the greater *sum of food* rather than the single best numeral. The second testing phase presented the monkeys with pairs of numerals in each arm, so that the total food rewards obtained in an arm was the sum of those two numerals. Each arm contained two numerals on every trial, always in the two most central locations within both arms (i.e., positions 2 and 3; every trial used the same four locations for numerals). There were four trial types with this presentation form (see Table 1 and Fig. 1e). The First Numerals Equal [Second Varied] trial type was the only one in which the total quantity difference of the arms was one or two items; the remaining trial types could each have a difference of one, two, or three items. Each monkey again completed 1,200 trials in this phase.

**Table 1:** Trial types used in Test Phase 2

Trial type	Description of correct arm contents	Example trial
First Numerals Equal [Second Varied]	Numerals in the first position of each arm are the same; correct arm choice depends on the numerals in the second position	**2 + 3**; 2 + 2
Second Numerals Equal [First Varied]	Numerals in the second position of each arm are the same; correct arm choice depends on the numerals in the first position	3 + 3; **4 + 3**
First Numeral Larger [Second Smaller] for Arm with Larger Total	The arm with the larger sum had a larger first numeral and a smaller second numeral compared to the other arm	**5 + 1**; 1 + 4
First Numeral Smaller [Second Larger] for Arm with Larger Total	The arm with the larger sum had a smaller first numeral and a larger second numeral compared to the other arm	4 + 1; **3 + 4**

Correct arm in bold

Note. For all trial types, the difference between sums in the two arms could be 1, 2, or 3 total items, except for First Numerals Equal [Second Varied], which could only have a difference of 1 or 2

*Test Phase 3 — Maximizing reward by comparing far, single numerals to pairs of numerals*. In this phase, the monkeys were shown three numerals placed throughout the maze. A single numeral was placed in one arm and two identical numerals were placed in the other arm. While it was randomized on each trial which arm would contain two numerals and which arm would contain only one, in this phase the positions of those numerals within the maze were fixed. The single numeral was always at a farther point from the start in its arm while one of the paired numerals was at a closer location to the start of the maze, and one was at the farther point. The arm with two numerals always contained the same numeral in both locations. In some trials, the combined value of the pair of numerals was greater than the single numeral in the other arm (e.g., 3 + 3 versus 5). Other trials were the opposite – the single numeral was larger than the sum of the two numerals in the other arm (e.g., 5 versus 2 + 2; see Fig. 1f). Five trial types were generated from this design (see Table 2). Each monkey completed 1,200 trials in this phase.

**Table 2:** Trial types used in Test Phase 3

Trial type	Description of arm contents	Example trial
Larger Total From Summed Larger Numerals	Presented two larger-value numerals with a single low-value numeral. Numeral value and the two-numeral array both were valid cues as to which arm offered the most reward	**4 + 4**; 2
Larger Total From Summed Smaller Numerals	Presented the largest numeral by itself and a pair of numerals in the other arm whose sum total was greater than that of the largest numeral. Numeral value was incongruent with largest possible total reward	**3 + 3**; 5
All Numerals Same	All numerals were the same; the only valid cue was which arm has the greater quantity of numerals in it	3; **3 + 3**
Lone Numeral Larger	Presented the largest overall value as a single number; this represented a larger reward than the combined value of the two smaller numerals in the opposing arm. Numeral value was the most important cue to use to maximize reward	1 + 1; **3**
Equal Sums	Presented a sum in each arm that was the same	4; 2 + 2

Correct arm in bold

*Test Phase 4 — Maximizing reward by comparing single numerals (in early or late positions) to pairs of numerals*. In this final phase, all aspects were the same as in Test Phase 3 except for the location of the numerals. Now, the single numeral could be in the closer or the farther position from the starting point within the arm. This allowed us to isolate and examine the role that distance to a numeral played in the monkeys’ choice behavior among the maze arms. In other words, this introduced the need for monkeys to include spatial distance and reward delay into their decision-making process along with trying to determine which arm would lead to the greater number of rewards. Monkeys again completed 1,200 trials in this phase.

## Results

*Training Phase 1 — Learning the value of numerals 1 through 5*. This phase simply required the completion of 30 trials of contacting the numeral in an arm as opposed to moving down the empty arm. Chewie and Murph did this in the minimum number of 30 trials, Lou required 31 trials, and Obi required 32 trials.

*Training Phase 2 — Learning to maximize reward by choosing the larger single numeral.* Monkeys now had to contact the larger numeral when one numeral was in each arm. Monkeys moved to the first test phase after successfully completing 41 of the last 50 trials (excluding the correction trials). Table 3 presents the total number of trials needed in this phase, the number of correction trials, and the overall percentage correct.

**Table 3:** Performance and number of trials completed in Training Phase 2 and Test Phase 1

	Prior experience?	Training Phase 2		Test Phase 1 % correct by difference			
		Trials to criterion (# Correction trials)	% Correct	1	2	3	4
Chewie	No	558 (180)	67.7	78.9	87.6	95.9	91.7
Lou	Yes	746 (319)	57.2	67.9	78.8	94.5	94.3
Murph	Yes	199 (63)	68.3	82.3	86.8	91.8	92.8
Obi	No	241 (76)	68.5	74.4	87.0	95.8	88.2

*Test Phase 1 — Comparing single numerals that vary in reward amount and distance*. Because the data were normally distributed, we conducted a repeated measures ANOVA with difference and trial type as the within-subjects factors and percentage correct as the dependent measure. Because Mauchly’s Test of Sphericity indicated that the assumption of sphericity was violated for the trial type variable, we used the Greenhouse–Geisser correction. The ANOVA indicated that there was a main effect of the difference between the two-numeral values, *F*(3, 9) = 13.16, *p* = 0.001, η^2^p = 0.81. There was not an effect of trial type, *F*(1.009, 3.028) = 7.12, *p* = 0.075, η^2^p = 0.70. There was not an interaction of difference and trial type, *F*(6, 18) = 0.86, *p* = 0.54, η^2^p = 0.23. Because there was only an effect of difference, the performance of the monkeys collapsed across trial type is shown for each difference in Table 3. The effect of numerical difference was reflected in a significantly positive correlation of difference and performance correct across all trial types, *r*(14) = 0.79, *p* < 0.001.

**Fig. 2:**
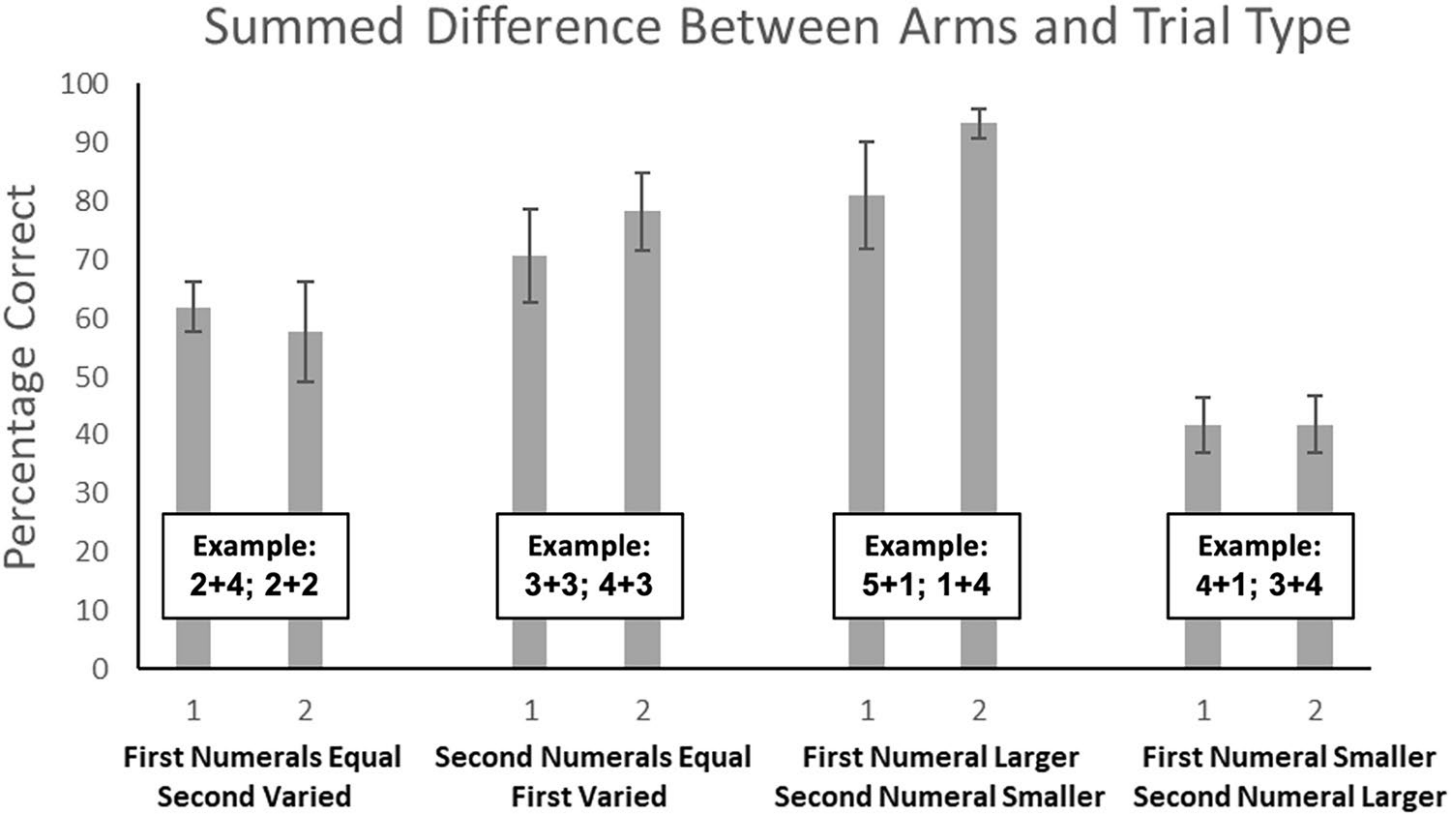
Performance of the monkeys in Test Phase 2, shown as a function of trial type and difference in rewards in the two maze arms. The x-axis number labels indicate the difference in quantity between the summed totals of the two maze arms. Error bars indicate 95% confidence intervals of the mean. Example trials are shown for each condition

*Test Phase 2 — Maximizing reward by comparing the sum of numerals*. For this phase, we examined performance as a function of trial type, and the difference between numeral values (Fig. 2). As noted in Table 1, for the trial type First Numerals Equal [Second Varied], only two differences in summed numeral values were presented, and so for the omnibus ANOVA test we only included differences of one or two in the summed total of the numerals in each arm. We found no significant effect of difference, *F*(1, 3) = 1.76, *p* = 0.28, η^2^p = 0.37. There was a significant effect of trial type, *F*(3, 9) = 60.15, *p *< 0.001, η^2^p = 0.95. The interaction also was significant, *F*(3, 9) = 4.29, *p* = 0.039, η^2^p = 0.59. To examine this interaction in more detail, we assessed performance separately for each trial type as a function of difference. We found no significant difference for the First Numerals Equal [Second Varied] trial type, *t*(3) = 0.70, *p* = 0.27, Cohen’s* d* = 0.35, the First Numeral Smaller [Second Larger] for Arm with Larger Total trial type, *t*(3) = 0.04, *p* = 0.49, Cohen’s* d* = 0.02, the Second Numerals Equal [First Varied] condition, *t*(3) = 2.47, *p* = 0.09, Cohen’s* d* = 1.23, or the First Numeral Larger [Second Smaller] for Arm with Larger Total trial type, *t*(3) = 2.76, *p* = 0.07, Cohen’s* d* = 1.38. This means that numeral difference was not related to performance in the two trial types in which using only the first numeral comparison to guide responding would lead to mistakes (i.e., First Numeral Smaller [Second Larger] for Arm with Larger Total and First Numerals Equal [Second Varied]). Difference also showed a nonsignificant relation to performance in the two trial types where the first numeral could be used to determine the arm with the greater sum of the numerals (i.e., Second Numerals Equal [First Varied] and First Numeral Larger [Second Smaller] for Arm with Larger Total).

To examine the effect of trial type, we compared each of the conditions to all others, collapsed across difference. This allowed us to use all data from this phase to see whether some trial types were more difficult than others. To account for the multiple tests, we used the Bonferroni correction to set alpha at 0.008. Performance in the Second Numerals Equal [First-Varied] condition was significantly better than performance in the First Numeral Smaller [Second Larger] for Arm with Larger Total condition, *t*(3) = 6.58, *p* = 0.007. Performance in the First Numeral Larger [Second Smaller] for Arm with Larger Total condition was significantly better than performance in the First Numeral Smaller [Second Larger] for Arm with Larger Total condition, *t*(3) = 10.94, *p* = 0.002. No other conditions differed from each other.

We also compared performance in each trial type to a 50% chance level using one-sample t tests. Three of four conditions indicated performance that exceeded chance levels, First Numerals Equal [Second Varied], *t*(3) = 3.34, *p* = 0.044, Second Numerals Equal [First Varied], *t*(3) = 6.91, *p* = 0.003, and First Numeral Larger [Second Smaller] for Arm with Larger Total, *t*(3) = 11.98, *p* < 0.001. However, performance in the First Numeral Smaller [Second Larger] for Arm with Larger Total condition was significantly below chance, *t*(3) = −4.59, *p* = 0.019.

We also examined overall performance as a function of whether the larger summed total was in the arm with the largest individual numeral (e.g., 4 + 1; 2 + 5; this was designated as a Congruent trial type) or if it was in the arm without that largest individual numeral (e.g., 3 + 4; 5 + 1; Incongruent trial type). This is important to assess to determine the degree to which the monkeys prioritized the largest numeral in a way that negated any effort to estimate the summed total of that arm (i.e., if they responded based on a bias to go for the single largest numeral). Overall, the group data indicated that there was not an effect of trial type (Congruent or Incongruent), *F*(1, 3), 6.51, *p* = 0.08, η^2^p = 0.68. There also was not an effect of difference, *F*(1, 3) = 5.36, *p* = 0.10, η^2^p = 0.64, and there was not an interaction, *F*(1, 3) = 3.61, *p* = 0.15, η^2^p = 0.55.

**Fig. 3:**
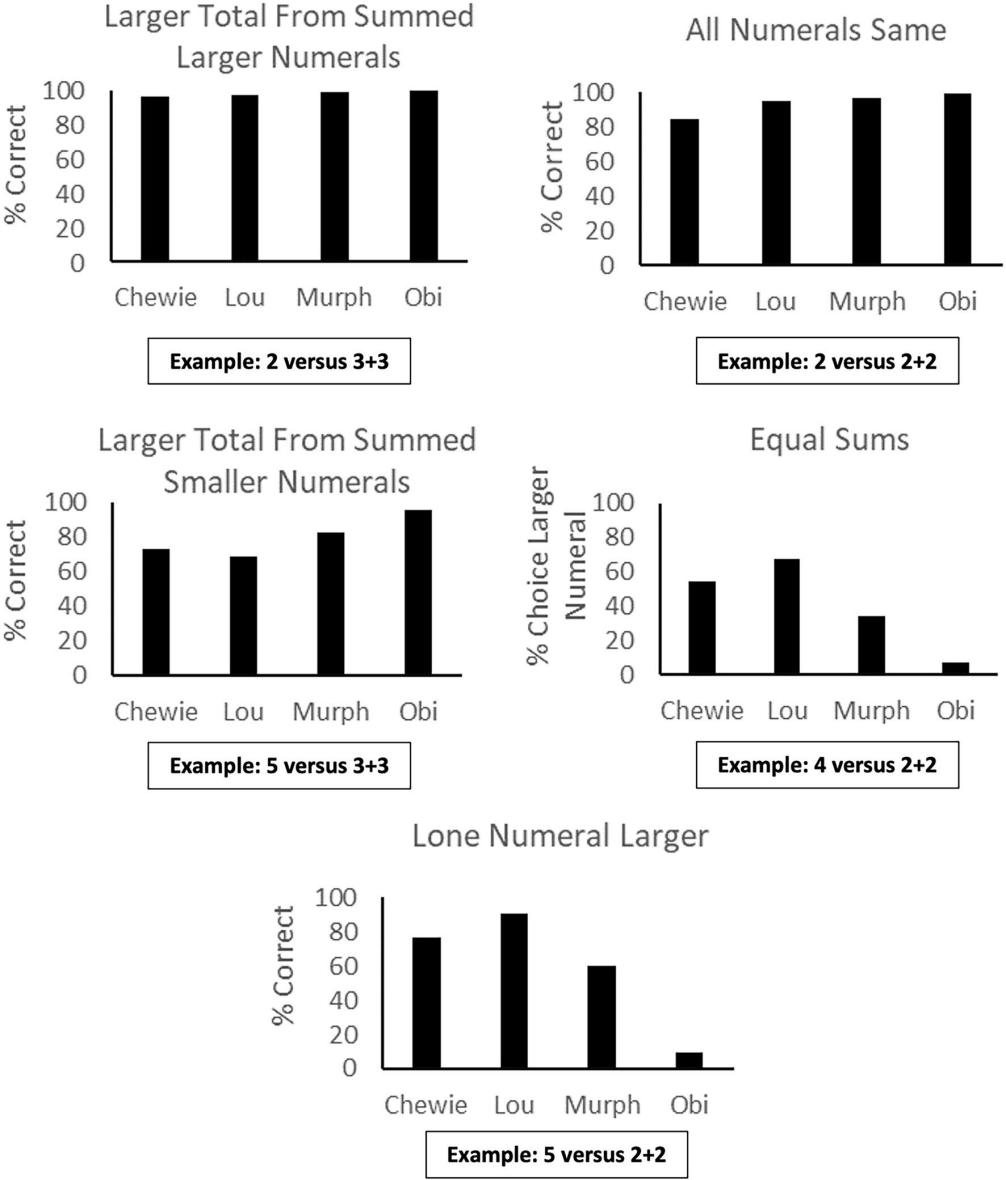
Performance of each monkey in Test Phase 3, shown as a function of trial type. Example trials are shown for each trial type

*Test Phase 3 — Maximizing reward by comparing far, single numerals to pairs of numerals.* The performance of the monkeys is shown in Fig. 3 for each trial type. Because each of these trial types assessed a specific aspect of behavior that we wanted to isolate, to determine its relevance in how the monkeys approached the task, we did not conduct any comparisons between trial types. Instead, we focused on group and individual differences from chance level, which would reflect indifference for that trial type. As expected, the group showed significantly above chance performance on the Larger Total From Summed Larger Numerals trial type, *t*(3) = 65.62, *p* < 0.001, Cohen’s *d* = 32.8. Also as expected, the group showed significantly above chance performance on the All Numerals Same trial type, *t*(3) = 13.73, *p* = 0.001, Cohen’s *d* = 6.86. For these two trial types, two-tailed binomial tests indicated that all monkeys were significantly better than chance, *p* < 0.001.

For the Larger Total From Summed Smaller Numerals trial type, which was anticipated to be more difficult, the group exceeded chance levels of performance, *t*(3) = 50.04, *p* = 0.015, Cohen’s *d* = 2.52, and all four monkeys were significantly better than chance, *p* < 0.001. For the Equal Sums condition, because there was no correct choice (both arms led to the same number of rewards), we examined the percentage of trials in which the arm with the largest individual numeral was chosen. The group did not differ from chance levels in choosing that arm, *t*(3) = 0.73, *p* = 0.26, Cohen’s *d* = 0.36. However, there were large individual differences in this trial type. Chewie did not differ from chance in his responding, *p* = 0.54, Lou showed a significant preference for the arm with the larger numeral, *p* < 0.001, and Murph (*p* = 0.003) and Obi (*p* < 0.001) showed a significant preference for the arm with two numerals of the smaller value. For the Lone Numeral Larger condition, the group did not differ from chance levels in choosing the arm with the greater total value (in this case, the arm with one numeral in it rather than two), *t*(3) = 0.52, *p* = 0.64, Cohen’s *d* = 0.26. However, at the individual level, Chewie, Lou, and Murph all showed a significant preference for that arm that provided more rewards, all *p* < 0.01, whereas Obi showed significantly below chance performance in this condition, *p* = 0.001. Thus, individual differences were clearly apparent in performance in this phase.

**Fig. 4:**
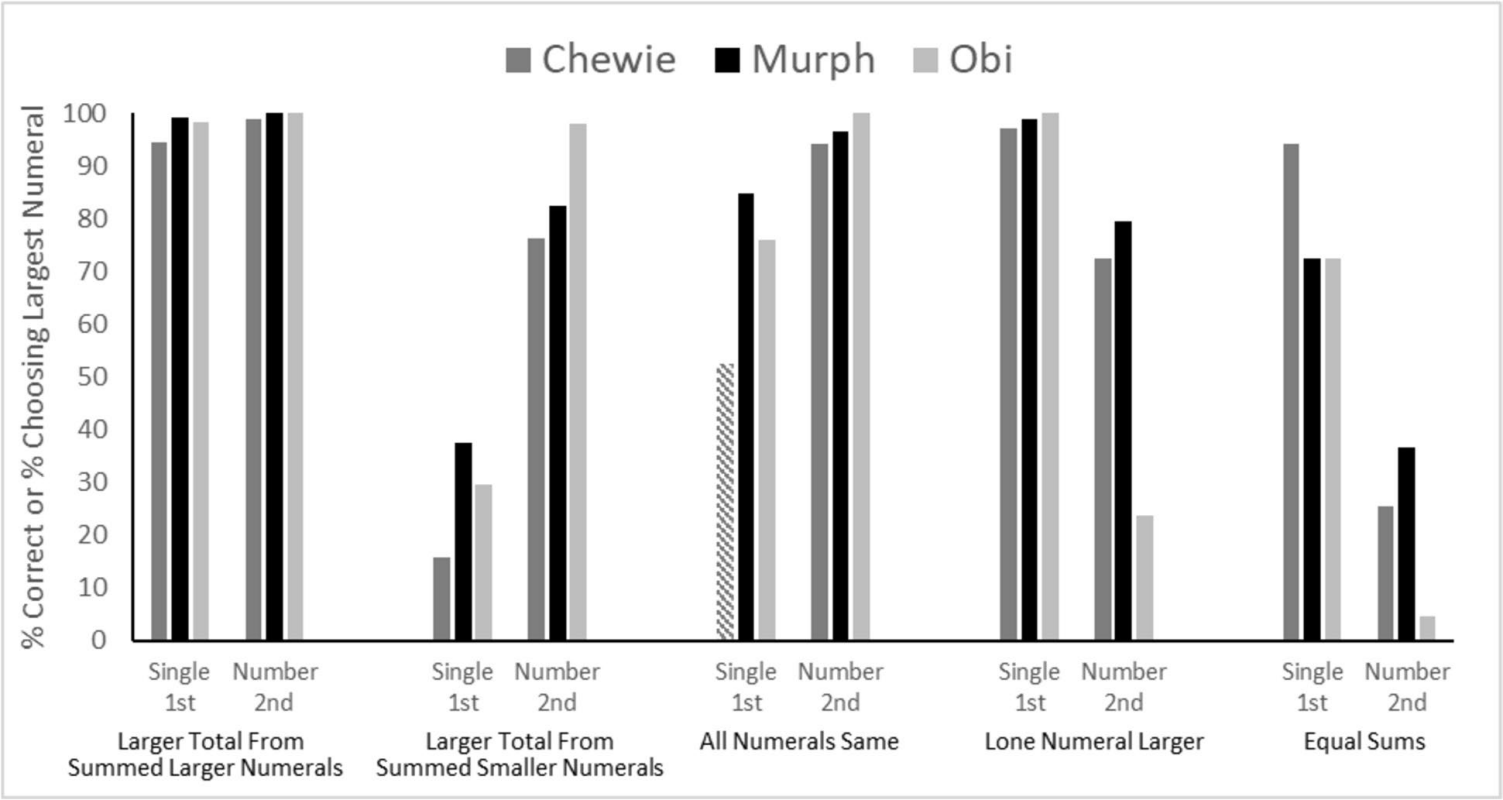
Performance of each monkey in Test Phase 4, shown as a function of trial type and which position the single number was located within the maze. The hatched bar indicates the one condition in which performance did not differ significantly from chance responding

*Test Phase 4 — Maximizing reward by comparing single numerals (in early or late positions) to pairs of numerals.* To best illustrate these data, we present choice behavior for each monkey in Fig. 4 for each trial type, as a function of whether the single numeral was early or late in the maze arm in which it was located. Data are first compared in each trial type/numeral location to chance level (50%). In all cases except one, all monkeys were either significantly above or significantly below chance levels of responding (all *p* < 0.05, binomial test). Figure 4 shows that for the Larger Total From Summed Larger Numerals trial types, it did not matter whether the single numeral was in the early or late position in the arm, and this is what one would expect given that the opposing arm had larger numerals and a greater total sum. For the Larger Total From Summed Smaller Numerals condition, position was an important factor. Monkeys performed much better (all above chance) when the single largest individual numeral was later in its arm than when it was earlier. In the earlier position, the monkeys were biased to move to that biggest numeral, even though the sum total of that arm was smaller than the other arm. This bias was the same for the Equal Sums. In this case, there was no correct answer, so this pattern was not detrimental to optimizing reward, but it again shows the prepotency of that largest numeral.

When both arms had the same numeral in them (and one arm had two such numerals), all monkeys performed above chance no matter whether the arm with only one numeral had it placed early or later in the arm. The one exception is that Chewie performed at chance levels when the single numeral was early (hatched bar in Fig. 4), suggesting that in these trials he was not discriminating the difference between one numeral of a given value, and two numerals of that same value. The other two monkeys did take into account the one-versus-two numerals as relevant, even when the numerals were, individually, all the same value.

Finally, in the condition in which the greater sum was in the arm with only one numeral (which necessarily also had to be the largest individual numeral shown on that trial), the monkeys performed very well, with one exception. Monkey Obi performed very poorly when that numeral was later in the arm (e.g., 2 + 2 in one arm, and a late 5 in the other arm). This again indicated that, for Obi, early numerals (that could be contacted more quickly) were more salient.

An important additional comparison is within each trial type looking at performance when the single number was either in the early spatial position or the later spatial position. To do this, we compared performance for each monkey in each condition using Fischer exact tests. For the Larger Total From Summed Larger Numerals, all three monkeys showed no difference in performance, all *p* > 0.08. For the Larger Total From Summed Smaller Numerals, all three monkeys showed significantly better performance when the single number was presented in the later position in the maze arm compared to the earlier position, all *p* < 0.01. For the All Numerals Same condition, this was also true, all *p* < 0.01. For the Lone Numeral Larger all monkeys were significantly better when the single numeral was presented in the earlier position, all *p* < 0.01.

## Discussion

Monkeys were trained to traverse computer maze arms to collect rewards based on the values of Arabic numerals they contacted. Learning that Arabic numerals represented different numbers of food rewards came fairly easily to the monkeys, which was unsurprising given their previous experience in computerized testing and past research showing that such numerals can be learned in their ordinal sequence (e.g., Beran et al. 2008; Harris et al. 2007a). Once trained, we then could assess how well the monkeys could distinguish how multiple numerals in each arm could lead to more or less food reward compared to the alternate arm.

Although traveling a greater distance in this task only delayed reward by a few seconds, when distance to a larger numeral was relevant, it did not disrupt the monkeys from maximizing reward (Test Phase 1). This result indicated another clear example of monkeys’ ability to wait a little longer, and “move” a little farther, for a better outcome, as has been shown in past work using computerized tasks (Evans 2007; Evans and Beran 2014; Evans et al. 2014) and assessing monkeys’ movement through space to different reward types (e.g., Stevens et al. 2005).

With the introduction of pairs of numerals, we began to see changes in performance. In Test Phase 2, in which two numerals were in each arm, the monkeys performed well overall, but there was also a clear bias to focus choices more on the difference in values of the first numeral in each arm, rather than the summed total. This suggested that the monkeys were specifically focused on the first quantity of food they could obtain (from the first numeral they touched). This makes sense given our design decision to provide pellets as soon as any numeral was contacted rather than after the entire arm had been traversed (we return to this point later). However, this bias was not absolute. In fact, despite performing more poorly in this trial type, the monkeys still opted for the greater total amount of food when presented with a smaller first numeral on about four trials out of ten, on average. This suggests that they sometimes recognized that the best total outcome was not going to come from choosing the better first numeral alone. Had their bias been stronger, performance would have been far worse, and likely well below chance levels on this trial type. In this way, the monkeys occasionally could overcome this bias to choose the single “best” item sometimes seen in other research. For example, chimpanzees sometimes showed a preference to choose one set of food rewards over another set when that first set had the biggest single item in it, even though the total amount of food in the set was smaller (Boysen et al. 2001). However, this bias also was not always evident in the choices of those chimpanzees, and they still sometimes selected the set with the overall greatest amount of food.

In Test Phase 3, the monkeys were presented with trials in which two numerals were in one arm, and one numeral was in the other arm, and at a farther distance from the start location. One trial type assessed how well monkeys could determine that a single numeral was more valuable than a pair of numerals even when the single numeral took longer to reach than the first numeral in the pair. Three of four monkeys showed a preference to travel longer to get to the single, largest numeral when that choice led to the most food rewards. One possible interpretation of this pattern was that monkeys were simply always choosing the arm that had the highest value numeral in it. This would be a pattern that was not reflective of attempting to sum numerals, but rather a strategy that was entirely focused on individual stimulus value. However, we found that the largest numeral was not the only relevant information they used. When the arm with two smaller numerals summed to a greater total amount, all monkeys then chose that arm. This result was perhaps most compelling for the claim that the monkeys could use individual numeral values, and a summative-like process to combine numeral values and estimate that that sum was greater than a numeral that, by itself, denoted the single largest reward amount. However, performance on those trials was aided slightly by the fact that the two-numeral arm also had the first numeral closer to the start location, so it was possible this spatial “benefit” was relevant for the monkeys. Test Phase 4 assessed this feature of the task by providing trials where the single number sometimes was early in spatial location in an arm and sometimes was later in spatial location. This allowed us to directly compare performance and see whether the good performances just noted were a result of confounding space with numeral value.

In Test Phase 4, we again saw a range of performances across monkeys, but some convergence toward conclusions about what was controlling behavior. Larger Total From Summed Larger Numerals trials, where the arm with a closer numeral also provided the greater total reward, were easiest, and all monkeys performed at very high levels no matter whether the single-numeral arm held its numeral closer or farther from the start location. When the arm with a single numeral represented the greater total amount (e.g., 5 versus 2 + 2), performance also was very high. However, for Larger Total From Summed Smaller Numerals trials, the role of spatial location became very important to performance. A single, large numeral, placed closer to the start location, often led to more errors in maximizing total reward because the other arm held more food (e.g., 3 + 3 versus 4 in the early position). All three monkeys tested in this phase were *below chance levels* on this trial type. Despite this condition indicating that the focus was only on the largest numeral presented, there was some secondary evidence that monkeys could attend to and use the number of numerals in the arms. When there was one numeral in one arm, and two of the same numerals in the other (e.g., 3 versus 3 + 3), the monkeys chose the arm with two numerals on the majority of trials. Most important to this issue is that two of three monkeys were above chance levels of performance even when the single numeral was in the early position. In those cases, the monkeys had to look beyond which of the two early numerals was larger because both were the same. They also had to recognize that one arm then had a second numeral later in the arm, that could lead to greater reward.

The role of spatial presentation again was evident in the trials in which total reward was equal in both arms (e.g., 4 versus 2 + 2). When the larger numeral was also in the closer position to the start location, monkeys significantly preferred to choose that arm. When it was at a greater distance, they preferred the arm with the two smaller numerals in it. This makes sense, given that these choices either led to all the food early in the run through an arm (e.g., choosing 4 over 2 + 2 when 4 was in the early location), or those choices led to food more immediately when the total was the same (e.g., eating two items early, then two items later, rather than four items later). These results also align with the idea that monkeys show a bias to choose numerals that they can reach more quickly, especially when that does not impact overall reward amount for the full trial duration.

Overall, the results of the experiment indicate that monkeys can sometimes use symbolic stimuli such as Arabic numerals to make decisions on a computer task. In many cases, they optimized reward, as when choosing between two numerals of different value or choosing between two pairs of numerals. More immediate reward versus delayed reward mattered and affected performance in those cases where a greater total could have been obtained, but only at a greater delay to getting the first rewards of the trial. That said, even in cases where a spatial bias existed, it was not absolute, and on some trials the monkeys still optimized reward despite this bias. Monkeys also sometimes prioritized the single largest numeral even when it was not in the arm of the largest total reward. This result matches what has been reported previously in our lab, with monkeys struggling to understand that symbol value and the number of symbolic stimuli in a choice set matter. In our past studies, as in the present study, symbolic stimuli representing single, large amounts of food reward often were over-estimated as to their contribution to the summed totals of sets (see Beran et al. 2005; Evans et al. 2010). This is interesting because it suggests some possible limits on whether animals can understand the requisite nature of combining quantities of symbols of value. This type of summation would be essential to being able to understand options in decision-making contexts like those used in assessing principles of behavioral economics. Of course, human children also go through the preschool years struggling with the idea that three 3s are worth more than one 6, as they are still mastering the ordinal sequence of the primary numeral symbol system (e.g., Brannon and van de Walle 2001; Colomé and Noël 2012; Hund et al. 2021). Thus, it is not surprising that this limitation may be robust in nonhuman species. It is typically not until after the age of 5 (and sometimes later) that children develop an intuitive (i.e., non-instructed) sense of multiplication (see Barth et al. 2009; McCrink and Spelke 2010). However, it would be important to assess whether there are contexts in which symbol value and symbol quantity are flexibly and accurately assessed and used in nonhuman animal choice behavior.

In summary, rhesus monkeys displayed the necessary self-control to travel farther in the maze (and wait longer) to receive larger rewards, although such delays were relatively short in terms of cursor travel time. Macaques sometimes overcame a bias to choose the single best item. While not always perfect (especially in Test Phase 2*)*, this requires the capacity to attend to more than just the single most salient stimulus present in a choice task to maximize reward. Monkeys were capable of summation of computerized numeral stimuli, indicating an understanding of the ordinal relations of such stimuli that allowed monkeys to estimate the relative overall value of multiple stimuli.

The use of a maze arrangement, with commitment to one arm that then had to be traversed, offers some clear benefits to this type of experimental question. Once animals know how to traverse the maze (i.e., that only one arm can be entered, and runs the length of the screen, etc.), one can confidently infer that stimuli presented within arms will impact why a participant chooses an arm, since they know they will contact those stimuli on that trial. This is easier to train than a visual sequence presented onscreen where there is less confidence that any arrangement (e.g., left to right, top to bottom) in open space will be interpreted to be a sequence to be encountered. Future use of this task could allow asking monkeys other kinds of questions about their preferences. For example, with some modification and more careful control for spatial arrangements, we could ask whether monkeys prefer to have rewards distributed in smaller, more frequent events, versus all of the rewards presented at the beginning, middle, or end of the trial (e.g., 2 + 2 + 2 + 2 + 2 versus 10 at once). At present, the literature is inconsistent on peak-end biases and other phenomena in animals, including monkeys, in which experiences must be considered as they occur in sequence (e.g., Blanchard et al. 2014; Xu et al. 2011). Future variations of this computerized maze task could add to the literature, and to other areas of the literature focused on decision-making processes in nonhuman animals.

It is important to note that in our current design, each numeral, when contacted, immediately presented rewards. We could have chosen to only provide the summed total of an arm with multiple numerals after both of those numerals were contacted. This would have dampened a bias to choose closer numerals if that bias was the result of wanted reward more immediately (which we assume any intelligent animal typically would prefer, all other things being equal). We predict performance might become more optimal when reward is always given only after full completion of the maze. Our present data suggest that monkeys would prefer the best outcome early in trials rather than later or dispersed throughout the trial, but that may not be accurate with more testing using these mazes. In the present design, however, monkeys exhibited some response inhibition difficulties (i.e., impulsivity) toward single, high valence stimuli in tasks where those stimuli must be considered in relation to overall values of choice sets as represented by summing the individual values of stimuli. Whether this is a difficult problem for them to overcome, or one that can be trained away or removed with more experience, remains to be determined.

## Data Availability

Data collected during this project will be made available upon request to the authors.
